# Effect of battery longevity on costs and health outcomes associated with cardiac implantable electronic devices: a Markov model-based Monte Carlo simulation

**DOI:** 10.1007/s10840-017-0289-8

**Published:** 2017-11-06

**Authors:** Jordana K. Schmier, Edmund C. Lau, Jasmine D. Patel, Juergen A. Klenk, Arnold J. Greenspon

**Affiliations:** 1 Exponent, Inc., 1800 Diagonal Road, Suite 500, Alexandria, VA 22314 USA; 2 Exponent, Inc., Menlo Park, CA USA; 3 Exponent, Inc., Philadelphia, PA USA; 4 Thomas Jefferson University, Philadelphia, PA USA

**Keywords:** Battery life, Cardiac resynchronization therapy devices, Costs and cost analysis, Device battery replacement, Device longevity

## Abstract

**Introduction:**

The effects of device and patient characteristics on health and economic outcomes in patients with cardiac implantable electronic devices (CIEDs) are unclear. Modeling can estimate costs and outcomes for patients with CIEDs under a variety of scenarios, varying battery longevity, comorbidities, and care settings. The objective of this analysis was to compare changes in patient outcomes and payer costs attributable to increases in battery life of implantable cardiac defibrillators (ICDs) and cardiac resynchronization therapy defibrillators (CRT-D).

**Methods and results:**

We developed a Monte Carlo Markov model simulation to follow patients through primary implant, postoperative maintenance, generator replacement, and revision states. Patients were simulated in 3-month increments for 15 years or until death. Key variables included Charlson Comorbidity Index, CIED type, legacy versus extended battery longevity, mortality rates (procedure and all-cause), infection and non-infectious complication rates, and care settings. Costs included procedure-related (facility and professional), maintenance, and infections and non-infectious complications, all derived from Medicare data (2004–2014, 5% sample). Outcomes included counts of battery replacements, revisions, infections and non-infectious complications, and discounted (3%) costs and life years. An increase in battery longevity in ICDs yielded reductions in numbers of revisions (by 23%), battery changes (by 44%), infections (by 23%), non-infectious complications (by 10%), and total costs per patient (by 9%). Analogous reductions for CRT-Ds were 23% (revisions), 32% (battery changes), 22% (infections), 8% (complications), and 10% (costs).

**Conclusion:**

Based on modeling results, as battery longevity increases, patients experience fewer adverse outcomes and healthcare costs are reduced. Understanding the magnitude of the cost benefit of extended battery life can inform budgeting and planning decisions by healthcare providers and insurers.

**Electronic supplementary material:**

The online version of this article (10.1007/s10840-017-0289-8) contains supplementary material, which is available to authorized users.

## Introduction

The use of cardiac implantable electronic devices (CIEDs) has been increasing in recent decades in the USA and worldwide [1, 2]. Both implantable cardiac defibrillators (ICDs) and cardiac resynchronization therapy defibrillators (CRT-Ds) have been associated with decreased mortality in patients with cardiomyopathy and heart failure associated with conduction delays [3]. In addition, patients with a history of life-threatening ventricular arrhythmias have improved survival with CIED implantation compared to antiarrhythmic drug therapy [4, 5]. The effectiveness of CIED implantation is tempered by the risks associated with the procedure. These risks include infections and non-infectious complications from the initial implantation or subsequent revision procedure [6–11]. Infection rates appear to be increasing faster than the rate of implantations [12, 13], only some of which can be explained by factors such as gender, comorbidities, or procedure complexity [1, 14]. It is well-understood that repeat procedures place the patient at higher risk for developing CIED infection [6, 9, 15]. Therefore, strategies to decrease the number of repeat CIED procedures are needed.

Device selection can be driven by many factors, including hospital policies and technology adoption practices [16, 17] and patient characteristics [18]. There are also studies directly comparing product performance and claims (e.g., von Gunten et al. [19], Zanon et al. [20]). A focus of several of these studies is on the increased longevity of selected CIEDs and the clinical and economic benefits associated with an extended device lifespan [21–23]. It stands to reason that longer device life would be associated with fewer pulse generator replacements and therefore costs should be lower, both because of fewer surgeries and the lowered risk and costs associated with surgical complications. There is also a tradeoff between battery longevity and maintaining the most contemporary technology in a given patient. In addition, patients who are candidates for ICD or CRT therapy typically have a number of significant medical comorbidities that affect their projected survival. A pulse generator with prolonged battery life would have little clinical value in a patient who is unlikely to survive until the time of elective pulse generator replacement. The case is not always clear-cut; however, clinicians need tools to help them with decision-making. They must balance the relative advantages and disadvantages of various features, including size, shape, lead technologies, and battery longevity as well as patient preferences. Yet, we are unaware of any studies that have explicitly modeled the impact of increased battery longevity on clinical outcomes and costs. Such models may be useful tools to guide physicians in making these clinical decisions with their patients.

The objective of this analysis was to compare changes in patient outcomes and payer costs attributable to increases in ICD and CRT-D battery life. The model characterizes complications and costs associated with CIED-related complications after a primary implantation. The model also includes a number of patient comorbidities, which affect overall survival. Analyses are specific to a patient cohort, a device type and battery length; comparisons reflect differences in costs and event rates experienced by patients for whom that device/battery was implanted.

## Methods

### Model structure

A state-transition (Markov) model was developed to estimate downstream health effects and payer-perspective costs following implantation of ICD and CRT-D devices. Model states represent defined phases of care (primary implantation, postoperative maintenance, revision, battery replacement) and an absorbing state (dead). Patients are simulated one at a time (i.e., at the patient level); patients face device procedure-related risks and mortality risks and accrue costs over a defined time horizon.

**Fig. 1:**
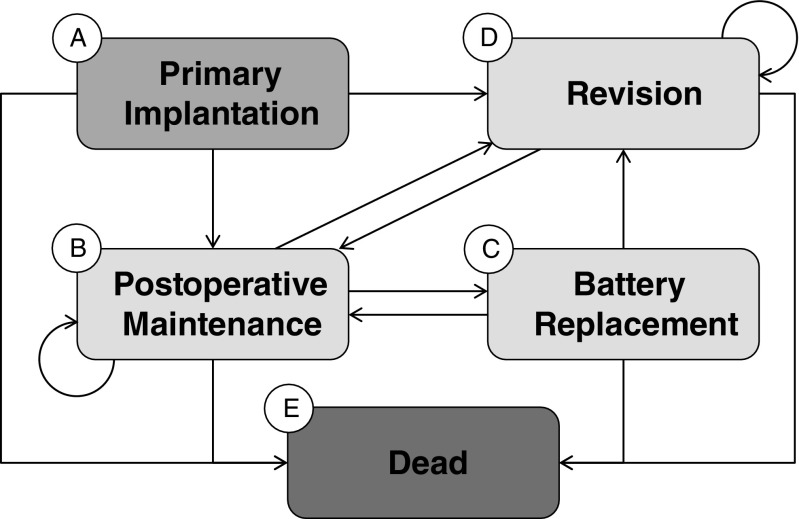
Markov state diagram

Figure 1 illustrates the ways in which patients flow through different paths in the model. The Markov states are indicated with letters; arrows indicate paths and transitions. Each transition has a probability associated with it; sources for probability values are presented in Table 1. These values were derived from an analysis of the Medicare data, using methods described in the “Input Data” section. Table 1 summarizes the high and low values for each of the scenarios. For example, the analysis identified the median and range of institutional and professional costs for the first quarter for patients who received an implant for inpatient and outpatient setting, for both device types, and for each of the three health categories (levels of the Charlson Comorbidity Index, 0–1, 2–3, and 4+). Each of these combinations was used in separate runs of the model, so that the model could be run only with inpatients with an ICD who had a Charlson Comorbidity score of 0–1 or patients who received a CRT-D as an outpatient and had a Charlson Comorbidity score greater than 4, for example. Some of these analyses are presented in detail in this manuscript; others are summarized. All patients enter the model at the primary implantation state (Markov state A). Following the implant, most patients proceed to the postoperative maintenance state (Markov state B); a small percentage requires a revision (Markov state D) in the next cycle. Most patients who enter the postoperative maintenance state remain there for multiple cycles. Battery replacements (Markov state C) can only happen after one or more cycles of postoperative maintenance, reflecting real-world patterns. Following a battery replacement, patients return to the postoperative maintenance state. Patients can remain in the same state for multiple cycles, indicated by loops, such as the loops shown on states B and D. “Dead” (Markov state E) is a terminal/absorbing state, meaning that there is no exit once patients have entered the state.

**Table 1:** Descriptions of input costs, proportions, and event rates

Category	Input	Stratified by^a^	Distribution (if appropriate) values	Comment/source
Quarterly costs	Primary implant	Setting Component Device type Charlson category	Lognormal Reference range, inpatient: Institution: $48,390–$52,243 Professional: $1168–$1517 Reference range, outpatient: Institution: $29,472–$32,077 Professional: $992–$1333	Derived from CMS data
	Revision	Setting Component Device type Charlson category	Lognormal Reference range, inpatient: Institution: $33,352–$49,522 Professional: $649–$939 Reference range, outpatient: Institution: $14,582–$21,199 Professional: $583–$747	Derived from CMS data
	Battery change	Component Device type Charlson category	Lognormal Reference range, outpatient: $24,054–$25,289	All battery changes were assumed to be performed on an outpatient basis
	Follow-up cycle	Device type Charlson category	Lognormal Reference range: $80–$164	Professional and institutional costs were summed
	Complication	Device type Charlson category	Beta Reference range: $896–$1150	Professional and institutional costs were summed
	Infection	Device type Charlson category	Beta Reference range: $23,817–$40,841	Professional and institutional costs were summed
Proportions	Case mix (Charlson categories)	Device type	N/A Reference case: 24–25% CCI 0–2, 56–58% CCI 3–4, 18–20% CCI 5+	Alternative tables provided for shifting patients to higher (sicker) or lower (healthier) categories
	Setting (inpatient or outpatient) of primary implant	Device type Charlson category	N/A Reference case: 56% ICD, 58% CRT-D inpatient	Alternatives provided to CMS defaults
	Setting (inpatient or outpatient) of revision	Device type Charlson category	N/A Reference case: 32–51% inpatient (depending on device and CCI)	Alternatives provided to CMS defaults
Event rates	Battery depletion	Device type Battery life Time since last generator change	N/A Reference case: Legacy, from CMS; Extended, Chelu et al. [24]	Averaged over all patients
	Revisions for other reasons	Device type Time since last revision	N/A Reference case: 0–2%, depending on device and CCI	Averaged over all patients
	Complications in 1st year after primary implant	Device type Charlson category	N/A Reference case: 4–7%, depending on device and CCI	
	Complications in 1st year after revision	Device type Charlson category	N/A Reference case: 6–8%, depending on device and CCI	
	Complications in 1st year after generator change	Device type Charlson category	N/A Reference case: 2–3%, depending on device and CCI	
	Infections in 1st year after primary implant	Device type Charlson category	N/A Reference case: 2–4%, depending on device and CCI	
	Infections in 1st year after revision	Device type Charlson category	N/A Reference case: 1–3%, depending on device and CCI	
	Infections in 1st year after generator change	Device type Charlson category	N/A Reference case: 3–7%, depending on device and CCI	
	All-cause mortality rates	Device type Charlson category	N/A Reference case: Based on age, derived from Saxon et al. [25]	
	Operative mortality rates	Surgical type	N/A Reference: primary implantation 1.7%, revision 3.4%, battery replacement 0.2%	

*CCI* Charlson Comorbidity Index, *CMS* Centers for Medicare and Medicaid Services, *CRT-D* cardiac resynchronization therapy implantable cardioverter defibrillator, *ICD* implantable cardioverter defibrillator, *N/A* not applicable

^a^Stratification categories: devices (ICD or CRT-D); Charlson category (0–2, 3–4, or 5+); setting (inpatient or outpatient); component (institutional or professional); surgical type (primary implant, generator change, or revision)

The model time horizon is fixed at 15 years and each cycle’s duration is one calendar quarter. An annual discount rate of 3% was applied to both total costs and life years to account for the net present value of healthcare costs. The model was designed and implemented using TreeAge Pro 2016 (TreeAge Software, Inc., Williamstown, MA) with an Excel graphic user interface available to control selected model inputs.

Simulating at the patient level allows for heterogeneity among patients and introduces history to the Markov states. Random variation across patients was introduced by simulating individual patients (Monte Carlo simulations) for each set of model parameters. The number of simulated patients per model run was 20,000, using a fixed seed to reduce individual variation.

### Input data

The primary source for model input data was de-identified administrative claims data (known as the Limited Data Set (LDS)) associated with the 5% sample of Medicare beneficiaries available from the Centers for Medicare and Medicaid Services (CMS). This 5% sample is equivalent to about 2.5 million Medicare beneficiaries. The LDS data files contain the healthcare service records from inpatient and outpatient encounters generated by these 2.5 million individuals. The data consist of seven components: institutional claims (inpatient, outpatient facility, durable medical equipment, home care, hospice, and skilled nursing facility) and professional claims (Part B). Beneficiaries in the 5% sample were systematically drawn and represent the broad spectrum of Medicare enrollees across age, gender, race, and geographic region. Each beneficiary was assigned a synthetic identification number, which allows longitudinal tracking of subsequent infection, revision, and other complications following the initial implant. Medicare claims were queried to identify beneficiaries with primary ICD or CRT-D implantations between January 1, 2004, and December 31, 2014. Primary ICD and CRT-D implantation was identified from Current Procedural Terminology (CPT) 33249. The concurrent presence of the CPT code 33225 suggested a CRT-D was implanted.

A full list of diagnosis and procedure codes that were used to identify primary implantation, revisions, battery replacements, and complications is provided in Supplemental Table 1. Model inputs for the reference and sensitivity analyses are provided in Table 1. Costs, transition rates between states (e.g., operative and other cause mortality rates), and event rates (e.g., rates of lead infection or dislodgement) were derived from the 5% sample. The time period from which data were derived varies. Transition rates and costs were derived using the entire period for which data were available (i.e., 2004–2014), although costs were inflated to January 2016 dollars. To reflect current trends in setting of care, the distribution of inpatient versus outpatient settings was derived from 2012 to 2014 data.

#### Patient and claim identification

In the retrospective Medicare claims analysis from which data were derived, beneficiaries entered the cohort continuously during the study period, starting on January 1, 2004, and were followed until the end of the study period (December 31, 2014), until they withdrew from Medicare or switched to a Medicare fee-for-service plan or until death. Beneficiaries were required to have been enrolled from January 1, 2003, with no study procedures during that year, to increase the likelihood that the first study procedure from 2004 forward was the patient’s initial CIED implant procedure. The beneficiary’s status was tracked using the matching 2004–2014 Medicare enrollment files, which provide annual age, resident state, entitlement status, date of death, and other enrollment information. Beneficiaries younger than 65 years old, those residing outside of the 50 US states, or those enrolled in Medicare-HMO programs whose claims were not submitted to CMS were excluded from this study. For each beneficiary in the cohort, healthcare service claims starting from the initial implant were extracted from the Medicare claims data, including claims for hospital stays, outpatient clinic visits, physician service, skilled nursing care, home health services, and hospice services. The overall health status of the beneficiary at time of the initial installation was characterized by the Charlson Comorbidity Index (CCI), a tool that uses comorbidities to describe wellness and to predict mortality and future healthcare resource use. Diagnoses and surgical procedures performed during this one-year pre-implant period were compiled for the calculation of the CCI.

Beneficiaries with CIED-related infections, either local pocket infection or systemic endovascular infection, were identified by either the ICD-9-CM diagnosis code 996.61 or any diagnosis code for cardiac device infection, fever, bacteremia, endocarditis, cellulitis, or sepsis. These diagnoses were required to be accompanied by a generator removal, system revision, lead revision, or pocket revision procedure on the same claim record in order to be identified as a device procedure-related infection. In addition to infection, other non-infection complications of interest include cardiac perforation, pneumothorax, cardiac arrest, pulmonary embolus, and hematoma. Supplemental Table 1 includes a full list of codes used to identify each procedure and complication. We calculated the quarterly risk of infection and non-infection complications following the primary installation. In addition, we also calculated the risk of infection and non-infection complication following a battery revision and following other types of revisions. Duration of battery life was classified as either “legacy,” which used data derived from the historical CMS claims data, or “extended,” for which data from a US-based registry from a manufacturer of an extended battery longevity CIED was used [24]. Although the model uses data for each quarter directly, the time until 50% of ICDs required revision was 30 quarters in Medicare data versus 52 quarters in the LATITUDE registry and 22 quarters versus 40 quarters for CRT-Ds in the Medicare and registry data, respectively. This distinction allowed us to analyze the impact of device longevity.

#### Cost analysis

We adopted a third-party payer perspective for this study. Cost is represented by the amount paid by the Medicare trust to hospitals, physicians, and other institutions for the care of these elderly cardiac patients. We calculated the institutional costs (hospitals, clinics, nursing facilities, and hospice) as well as professional costs from physicians and other medical professionals. Quarterly costs, aggregated into two categories (institutional and professional), were calculated for patients with a non-zero cost. All costs were adjusted to the January 2016 level by the Consumer Price Index for medical care services published monthly by the US Bureau of Labor Statistics. Part D (drug costs) and costs that were the patient’s responsibility to pay out of pocket were unavailable and were excluded. We calculated cost of the primary installation, the cost associated with infection and non-infection complications, as well as quarterly “maintenance” cost (CPT: 93289, 93282, 93283, 93284, 93295, 93296, 93287) associated with the monitoring and periodic service of the CIED devices.

To capture the typical skew in costs and avoid assignment of negative costs, we assumed costs (with some exceptions; see Table 1) followed a lognormal distribution. Separate professional and institutional costs (with some exceptions; see Table 1) stratified by device and CCI category were drawn every cycle and accrued to patients experiencing events.

### Model assumptions

The complexity of the model required a number of assumptions. These are provided in more detail in Supplemental Table 2.

### Scenarios and analyses

Setup of the model begins with specifying scenario variables. The reference analysis requires the following parameter settings:

- Device type: ICD or CRT-D devices (run separately)
- Battery lifetime: lifetimes based on CMS data (“legacy”) versus an “extended” lifetime (run separately)
- Charlson Comorbidity Index: distribution of CCI based on CMS datasets
- Mortality rates from procedures: Default values based on CMS datasets
- Procedure settings: The inpatient/outpatient ratio for primary implantation and revision based on CMS datasets
- Cost data: based on CMS datasets, inflated to January 2016

Sensitivity analyses examined include a sicker (average higher CCI) population and an average of 20% more outpatient procedures as well as discounting costs and outcomes at rates of 6% and 0% annually to address the lack of consensus on appropriate discounting rates for health economic studies [26, 27].

### Model outputs

Patient-level event counts, costs, and life years are generated for each model run, specific to a device and battery length. Key outputs of interest include decreases in complications and costs with increasing battery life and numbers of patients requiring generator changes or revisions over the 15-year time horizon (or remaining lifespan).

## Results

Among the ICD and CRT-D recipients identified in the CMS database, mean age was 74.6 (standard deviation 6.1 years) and 73.3% were male. Across all beneficiaries, approximately one quarter had a CCI score of 2 or less and more than half had a CCI of less than 5. There were no significant differences in age, sex, or CCI score between ICD and CRT-D patients.

**Fig. 2:**
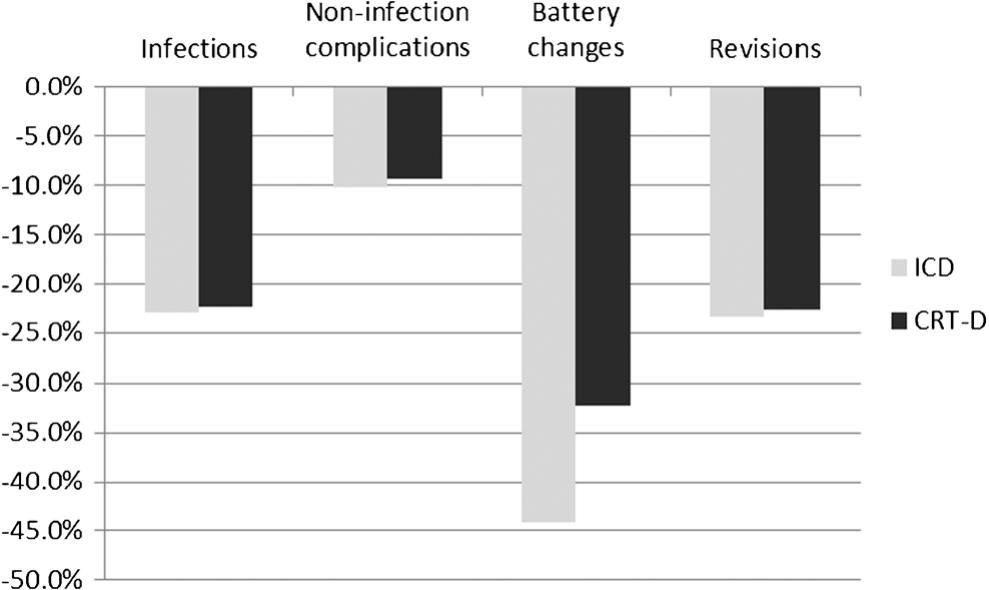
Change in repeat events, legacy versus extended longevity by device

**Fig. 3:**
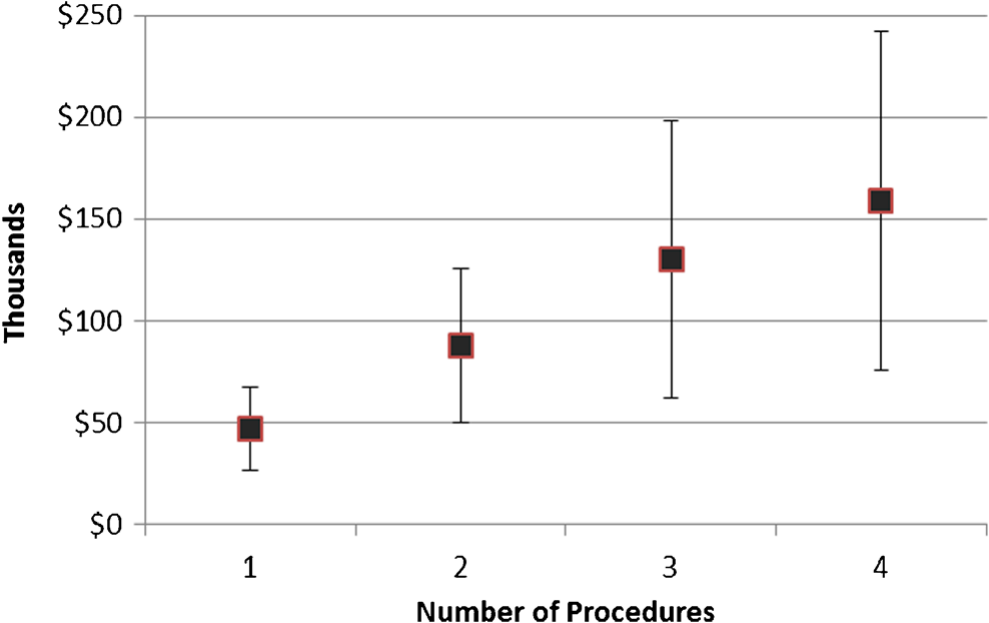
Mean discounted costs by number of repeat procedures, ICD legacy longevity scenario

Patients receiving ICDs with a longer battery life were more likely to have no generator changes (82.2 versus 68.1%), no infections (94.3 versus 92.6%), no non-infection complications (91.2 versus 90.2%), and no revisions (94.1 versus 92.3%, Table 2) during the modeled period. The relative reduction in each outcome is shown in Fig. 2. Patients receiving CRT-Ds with a longer battery life experienced similar additional benefits (Table 2). As expected, patients with additional procedures incurred greater costs, as shown for the ICD legacy scenario (Fig. 3); the same pattern was shown for other devices and longevity options. A longer battery life was associated with a decrease in total costs for patients with both ICDs and CRT-Ds (Table 2) and practically equivalent life years. Small increases in life years were observed with longer battery life, but as they were consistently less than 1 month of the 15-year model horizon and therefore less than one model cycle, they are treated as equivalent. Patients receiving ICDs with an extended battery had total costs reduced by approximately $5288 (9%) compared to the legacy battery, while patients receiving CRT-Ds with an extended battery had total costs reduced by roughly $5981 (10%). Cost-effectiveness ratios are not defined for extended battery strategies because they are cost saving, that is, extended batteries are associated with better (lower) costs and better (nominally increased) life years outcomes.

**Table 2:** Clinical and cost results, percentages of patients with no adverse events or repeat procedures, costs, and life years

Device and battery length	With no infections (%)	With no non-infection complications (%)	With no battery changes (%)	With no revisions (%)	Neither battery change nor revision (%)	Total costs (mean, SD)	Life years (mean, SD)
ICD, legacy	92.6	90.2	68.1	92.3	65.0	$56,384 ($32,385)	6.53 (4.64)
ICD, extended	94.3	91.2	82.2	94.1	78.5	$51,096 ($28,565)	6.54 (4.65)
CRT-D, legacy	89.7	91.5	61.9	89.4	58.6	$62,930 ($36,599)	5.50 (4.29)
CRT-D, extended	92.0	92.3	74.2	91.8	70.1	$56,949 ($32,091)	5.51 (4.28)

Costs and life years discounted at 3% annually

*CRT-D* cardiac resynchronization therapy implantable cardioverter defibrillator, *ICD* implantable cardioverter defibrillator, *SD* standard deviation

**Fig. 4:**
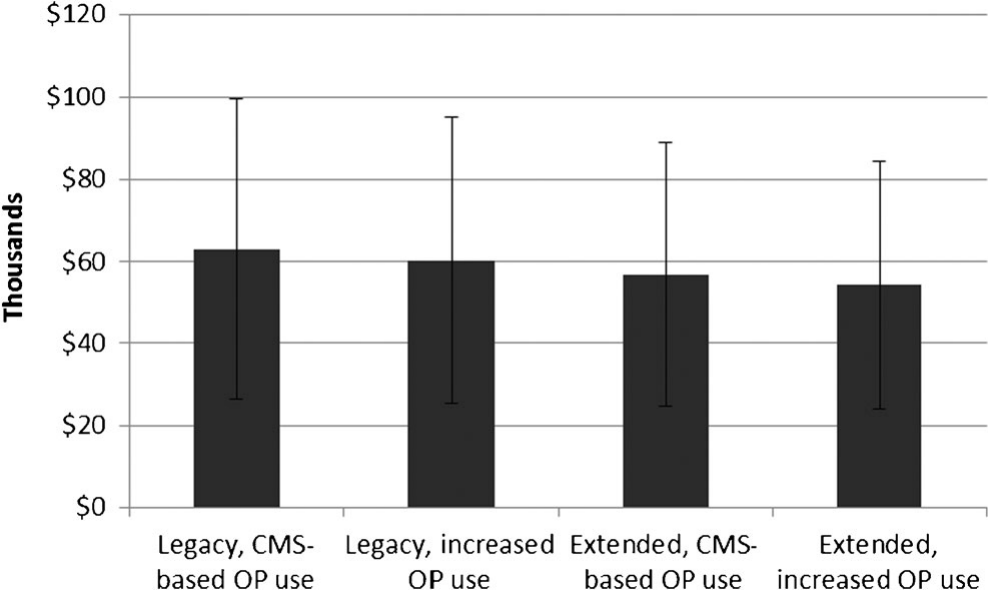
Sensitivity analysis: change in total costs by longevity and shift to outpatient procedures, CRT-D only

Sensitivity analyses both confirmed the model’s behavior and provided additional insight. The following examples reflect patients with ICDs, although similar patterns were evident among CRT-D patients. For example, patients who were sicker, operationalized as shifting the Charlson Comorbidity Index, showed the same pattern, with costs for the existing battery life greater than an extended battery life, but with lower costs for each scenario to reflect earlier death. The total discounted life years accrued were almost a full year less than in the base case scenario. Patients who were healthier and had lower CCI scores tended to live longer, but repeat procedure rates were similar to the reference CCI distribution. This is likely because living longer creates more opportunities for the need for battery revisions. Sensitivity analyses also explored the impact of having 20% more implant and revision procedures performed in outpatient settings. (All battery changes were assumed to be performed in outpatient settings.) There was no difference in life years accumulated, as the model did not assign different mortality rates based on procedure setting, but costs for the legacy and extended battery scenarios were approximately $2600 less than the base case setting distribution. Figure 4 shows costs when using the observed proportion of outpatient implant and revision procedures for the legacy and extended battery scenarios for CRT-D devices using CMS data on care setting compared to increased use of outpatient care (increasing, for example, the percent of initial ICD implants performed in outpatient settings from 56 to 76%). This more accurately reflects recent trends in care settings. Increasing the ratio of outpatient to inpatient procedures lowered costs by 4–5% over the modeled observation period.

**Fig. 5:**
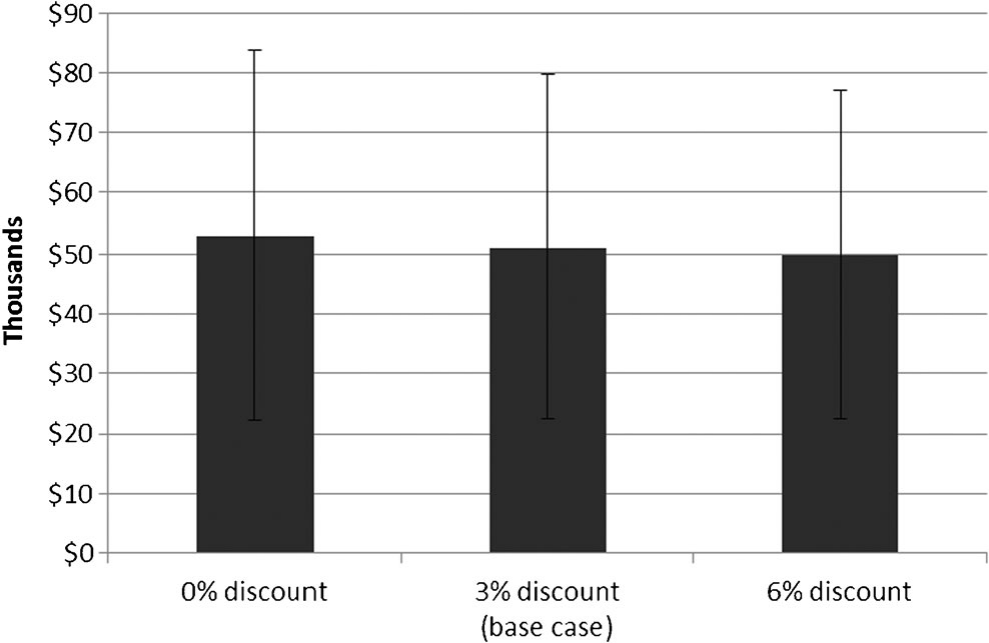
Sensitivity analysis: effect of discounting on total costs, ICD extended longevity scenario

Other sensitivity analyses show the effect of changing input variables. Figure 5 demonstrates how changing the discount rate affects total costs with extended longevity ICD devices. As shown, there is a small but expected decrease with a higher discount rate and an increase with no discount rate. It is not surprising that the discount rate only changes costs slightly; most of the cost for each patient is typically in the first cycle, with the initial implant.

## Discussion

This analysis found that increases in battery life substantially reduce costs and certain unwanted events (infections, generator changes, revisions) and produce equivalent or minimal gains in life expectancy. Extended battery life was the dominant strategy for all examined scenarios. While this finding is consistent with expectations, we believe that presenting empiric evidence of the value of increased battery life can serve multiple purposes. First, the typical evidence stream from randomized controlled trials does not exist for battery components, yet some alternative evidence is often required to demonstrate value. Second, understanding the shift in costs that would be associated with extended battery life can be used for budgeting and planning. Finally, while understanding cost savings associated with longer battery life is valuable for estimating budget impact of CIED use for insurers, estimating repeat hospitalizations can also have value to facilities and healthcare systems in planning inpatient care. For these reasons, understanding the magnitude of the cost benefit of extended battery life warrants analysis rather than assumptions. The UK’s National Institute for Health and Care Excellence (NICE) has already considered this question; it developed and issued guidance on its support for a CRT-D with extended battery life based on published evidence [28]. The USA currently employs a fee-for-service approach to healthcare, which predominantly rewards for volume of care. As the USA explores alternative payment models with greater emphasis on quality outcomes and value of care, it will be particularly important to provide evidence on the economic value of medical therapies and their differentiating properties.

Pulse generator replacements due to battery depletion have typically occurred between 4 and 6 years after ICD implantation [29]. The problem of battery longevity is not new; over the years, multiple factors have contributed to the long-standing observation that battery life is insufficient for the post-implantation survival [30] including trends toward miniaturization, earlier use, and broadening of indications [31]. Further complicating these factors is the pace of innovation. ICD patients may be upgraded to different or more advanced devices before batteries fail or without the presence of a complication. Patients who received ICD upgrades rather than revisions or generator replacements were not excluded from the present analysis. Later review determined that they comprised less than 4% of ICD “revision” procedures and no adjustments were made. Dual-chamber devices may be associated with higher short-term complication rates [32], and it is possible that patients who received upgrades may be clinically different than others, but there were insufficient patients to test in this population.

Repeated revision procedures have also been identified as a risk factor for CIED complications, in particular, infection [6, 9, 33]. Unfortunately, the rate of CIED infection appears to be increasing out of proportion to overall device utilization [12–14]. Infection rates may be influenced by multiple factors, including patient comorbidities, but also by many other factors that are not detectable with a claims database [34]. Further refinement of the model could permit incorporating some of the factors, although there are practical limits to what factors can be considered based on the data set and counts of patients in relevant categories. While reducing the need for revisions would not eliminate all infections, as a primary driver of infection, any reduction in revisions would be noteworthy.

From a clinical perspective, longer battery life with consistent performance seems like an obvious step forward. Payers must weigh clinical benefits against budget implications and are increasingly cognizant of cost-effectiveness of treatment and long-term patient outcomes. Hospitals have even more of a stake in patient success including reducing readmissions than in previous years, as competition for demonstrating quality through improved patient outcomes converges with penalties for unnecessary readmissions. Studies like the model presented here can provide value to multiple audiences. They can help third-party payers consider coverage, assist hospitals in predicting caseload (revisions versus implantations), and potentially help patients who may have a co-pay evaluate how they may benefit from any increased cost to which they may be subject based on battery and device type. Still, it is not logical to cover a device that will long outlast a patient’s lifetime; models like this one can help explore in what scenarios increased battery longevity has the greatest impact on different populations.

### Limitations

This model was designed to allow the user to account for changes in the setting of implant and revision procedures, which can have a substantial impact on costs, and across levels of patient health, as measured by the Charlson Comorbidity Index. The Charlson Comorbidity Index used for stratifying patients did not explicitly account for patient age, but alternative comorbidity indices could be explored, which would permit correlation of patient age to event rates and mortality risks, as well as costs. In particular, while the age of patients is constrained by the use of the Medicare claims data, there are other clinical factors for which we cannot account using an administrative claims database. Specifically, we do not know about the frequency of pacing or ICD shocks, which could offer substantial insight into battery performance. The administrative nature of the database means that our ability to identify comorbidities and other health-related characteristics is limited to those for which a claim and diagnosis code are present. Although claims have been adjudicated by the time the dataset is available for analysis, the coding may not always be complete. For example, it is possible that there are codes that are not listed because there is no additional reimbursement for them. This analysis, consistent with most claims analyses, assumes that all relevant codes have been submitted as part of each claim. Other factors that were not included in this model may affect outcomes. For example, patient demographic and clinical characteristics, activity levels, battery consumption, and device characteristics may affect outcomes but are not available in a claims database. The model can be modified to include additional parameters as more information becomes available. Similarly, although the model only attributes infections that occur in the first year following a generator change, the model’s framework could be used to consider infections that occur over shorter or longer follow-up periods. The one-year window has been used in other CIED claims analyses [35]; longer time periods create greater uncertainty about attribution.

Another important limitation to consider is that this model used data from Medicare beneficiaries age 65 and older. In fact, the Medicare population from whom model input parameters were drawn averaged 74 years old. Selecting only the youngest Medicare beneficiaries with CIEDs would not have provided sufficient counts. Older patients may have fewer years in which to benefit from the CIED, so limiting the input parameters to data from older patients might constrain variation in life expectancy and thus minimize benefits. Recent analyses suggest that the average age at implant of an ICD in the USA is 67 ± 13 years, with one fourth of new implants in patients 59 years or younger [36]. Data from younger patients, who could also have fewer comorbidities, could reasonably demonstrate a greater benefit from the use of devices with longer battery lives. Alternatively, these patients receiving CIEDs earlier might be less healthy than those who do not require a device until they are older. Younger patients might be more likely to be in the workforce and have reduced work productivity associated with negative outcomes related to battery life.

Another concern about the use of the Medicare population for the input parameters and “legacy” battery longevity is that the population in the registry that supplied the “extended” longevity data may differ. It is likely that the Medicare population is more heterogeneous, but the possibility of younger patients in the registry may also affect heterogeneity. It is unclear how much overlap there may be between the Medicare population and the sponsor-initiated registry, as well as whether there are other differences between patients who participate in registries and those who do not.

The model is conservative in estimating the impact of implantations and revisions. It does not include either quality of life or societal costs or incorporate patient preferences, which suggest that patients value longevity over size [37]. Caregiving associated with implantable cardiac devices has been shown to have an impact on the well-being of patients’ partners that could translate to an increased health care burden among caregivers [38, 39]. Further, loss of work or other types of indirect costs associated with battery replacements and related adverse events can only increase total economic burden.

## Conclusion

Based on modeling results, as battery longevity increases, patients experience fewer adverse outcomes and healthcare costs are reduced.

## Electronic supplementary material

ESM 1 (DOCX 35 kb)
