# Supplementary material for: Effect of battery longevity on costs and health outcomes associated with cardiac implantable electronic devices: a Markov model-based Monte Carlo simulation
Source: J Interv Card Electrophysiol. 2017 Nov 6;50(2):149–58. doi: 10.1007/s10840-017-0289-8 (PMC5705743; doi:10.1007/s10840-017-0289-8)
Supplement: Supplementary file 1 — (DOCX 35 kb) [file 10840_2017_289_MOESM1_ESM.docx]

**Supplemental Table 1. Codes Used to Identify Procedures and Complications**

| Category | CPT or ICD-9 Procedure Code(s) |
| --- | --- |
| Battery only revision | Claim with (33240 or 33230 or 33231) and 33241 but without (33249 or 33225)  Claim with (33262 or 33263 or 33264) but without (33249 or 33225) |
| Other types of revisions | Claims with the following codes that do not meet the above criteria for “battery only revision:”  33230-33231  33241  33243  33244  33215  33216  33217  33218  33220  33223-33226  33262-33264 |
| Complications, infection | Septicemia (038)  Endocarditis (421.0, 421.9, 424.9)  Cellulitis (682.2, 682.9)  Fever (780.6)  Shock (785.52, 785.59)  Bacteremia (790.7)  Infection due to device (966.61) |
| Complications, non-infection | Chest tube (ICD procedure code 34.04)  Periocardiocentesis (ICD procedure code 37.0)  Pulmonary embolism (415.1, 415.11, 415.19)  Hemopericardium (423.0)  Cardiac tamponade (423.3)  Unspecified disease of pericardium (423.9)  Pleural effusion (511.8, 511.9)  Pneumothorax (512.0, 512.1, 512.8)  Respiratory arrest (799.1)  Hematoma (998.1) |

Note: Codes are CPT codes

**Supplemental Table 2. Model Assumptions**

| **Assumptions related to costs of primary implants, revisions, and generator changes:** |
| --- |
| - Primary implant costs (drawn from lognormal distributions every cycle) varied by setting (inpatient vs. outpatient), device, and Charlson category. Professional and institutional costs were modeled separately. The additional cost of managing infection was accounted for separately (see below). - Revision costs (drawn from lognormal distributions every cycle) varied by setting (inpatient vs. outpatient), device, and Charlson category. Professional and institutional costs were modeled separately. Costs were assumed to be the same for revisions stemming from infection and revisions stemming from other causes. The additional cost of managing infection was accounted for separately (see below). - For patients whose institutional (aka facility) costs of a revision (drawn from a distribution) exceeded $15,000, we assumed the full device was changed and set the counter for time since battery change to 0, and the time since a revision to 0. Otherwise, the less expensive patients are assumed to have only lead changes and the counters for battery changes and revisions are left unchanged, thus leaving them open to the possibility of having a battery change as described below. - Generator change costs (drawn from lognormal distributions every cycle) varied by device and Charlson category. Professional and institutional costs were modeled separately. Generator changes were assumed to be performed on an outpatient basis. The additional cost of managing infection was accounted for separately (see below). |
| **Infection- and non-infection complication-related assumptions:** |
| - Infection leads to additional costs in current cycle and revision in subsequent cycle. - Non-infection complication leads to additional costs in current cycle but does not lead to revision in subsequent cycle. - Infection and non-infection complication risk was tied to the most recent procedure (primary implant, battery only, or revision) and varied by device and CCI category. - Infection and non-infection complication costs (drawn from beta distributions every cycle) varied by device and Charlson category, but did not vary by the most recent procedure. Professional and institutional costs for infection and non-infection complication were not modeled separately. - Rates of infection and of non-infectious causes of revision are derived, not taken directly from CMS data, due to concerns over double-counting. - Assumed there were no infection-caused revisions more than 1 year after an implant. |
| **Assumptions related to mortality:** |
| - From CMS data, the 30-day mortality (all-cause) was used to estimate a probability of operative mortality, via a weighted (by distribution of patients across Charlson categories) average, for an estimate of 1.69% for the first 30 days after primary implant. Corresponding values for operative mortality for post-revision were 3.38% for revisions (double the probability of primary implant) and a low value of 0.2% for generator changes only. Operative mortality rates did not vary by device. - Extrapolating mortality rates (stratified by device and Charlson category) past observed CMS data was required to model a 15-year time horizon. A linear (exponential) fit to observed values was used to estimate (smoothed) rates. |
| **Assumptions related to follow up costs:** |
| - Costs of follow-up (post-implantation) are as observed in CMS data, by device and Charlson category. |
| **Assumptions related to generator changes beyond 1 year after implant:** |
| - We assumed that the decrease in rates of generator-only change after primary implant observed in CMS claims data was an artifact of patients dropping out of the risk pool due to having had a revision (which includes a generator change) or a generator change alone. Therefore, we held constant the maximum rate. - Generators were either classified as “legacy” battery length, which was derived from an analysis of CMS data, or “extended” battery length, which was derived from an ongoing registry.[24] - Because rates for battery depletion exhibited no consistent ranking by CCI category, we used overall rates (i.e., averaged over the 3 CCI categories, by device). |
